# Cutaneous Clues in Kawasaki Disease: Clinical Implications and Differential Diagnosis with Multisystem Inflammatory Syndrome in Children

**DOI:** 10.3390/jcm15031126

**Published:** 2026-01-31

**Authors:** Federico Carlini, Ada Marcella Chiesa, Martina Verzina, Chiara Sassetti, Donato Rigante, Susanna Esposito

**Affiliations:** 1 Pediatric Clinic, Department of Medicine and Surgery, University of Parma, 43126 Parma, Italy; federico.carlini@unipr.it (F.C.); adamarcella.chiesa@unipr.it (A.M.C.); martina.verzina@unipr.it (M.V.); chiara.sassetti@unipr.it (C.S.); 2 Department of Life Sciences and Public Health, Fondazione Policlinico Universitario A. Gemelli IRCCS, 00168 Rome, Italy; 3 Università Cattolica Sacro Cuore, 00168 Rome, Italy

**Keywords:** Kawasaki disease, multisystem inflammatory syndrome in children, SARS-CoV-2, cutaneous manifestations, pediatric vasculitis, mucocutaneous involvement

## Abstract

Kawasaki disease (KD) and multisystem inflammatory syndrome in children (MIS-C) are pediatric inflammatory conditions with overlapping mucocutaneous features that may complicate early diagnosis. We performed a narrative review of the literature to characterize and compare cutaneous manifestations reported in children with KD and MIS-C and to assess their diagnostic relevance. Published studies describing dermatologic findings in patients aged 0–18 years were reviewed. The analysis revealed a broad heterogeneity of skin manifestations in both conditions, ranging from classic polymorphous rash and acral erythema to atypical presentations, including annular, psoriasiform, vesiculobullous, urticarial, and erythema nodosum-like lesions. Reactivation at *Bacillus Calmette–Guérin* vaccination sites and associated mucocutaneous findings, such as conjunctivitis and oral changes, emerged as supportive diagnostic clues, particularly for incomplete KD. Considerable overlap in cutaneous phenotypes between KD and MIS-C was observed, especially in patients with persistent fever and systemic inflammation, highlighting the risk of diagnostic delay. These findings underscore the importance of recognizing atypical dermatologic patterns as part of an integrated diagnostic approach, as delayed identification may increase the risk of cardiovascular complications. Early recognition of cutaneous clues can support timely initiation of immunomodulatory therapy and improve clinical outcomes.

## 1. Background

Cutaneous manifestations are frequent and often early features of both Kawasaki disease (KD) and multisystem inflammatory syndrome in children (MIS-C). Although these conditions differ in epidemiology and triggers, they share overlapping clinical features and immune-mediated pathogenic mechanisms, making early recognition and differential diagnosis challenging [1]. Dermatologic findings are particularly relevant, as they may represent the first clinical clue and precede severe systemic complications.

KD is an acute vasculitis of unknown etiology that primarily affects children under five years of age [1,2]. Delayed or inadequate treatment is associated with coronary artery abnormalities, including aneurysm formation and myocardial ischemia [3,4]. Diagnosis is based on persistent fever and a constellation of clinical criteria, among which cutaneous manifestations—such as polymorphous rash, acral erythema or edema, and periungual desquamation—are prominent but nonspecific [5,6]. Consequently, atypical or incomplete presentations may delay diagnosis and increase the risk of cardiac involvement [7].

MIS-C emerged during the COVID-19 pandemic as a post—SARS-CoV-2 hyperinflammatory syndrome affecting a small proportion of infected children [7,8]. It is characterized by persistent fever, laboratory evidence of inflammation, and multisystem involvement, frequently including the cardiovascular, gastrointestinal, and mucocutaneous systems. Current evidence supports an immune-mediated pathogenesis driven by post-infectious immune dysregulation, molecular mimicry, and exaggerated cytokine responses [9,10,11]. Cutaneous findings occur in approximately 70–75% of cases and display wide morphological variability, ranging from morbilliform and scarlatiniform rashes to urticarial and reticulated patterns [11]. This heterogeneity, combined with substantial overlap with KD-related skin findings, further complicates early diagnostic differentiation [10].

Despite the growing number of descriptive reports on mucocutaneous findings in KD and MIS-C, a clear evidence-based framework that contextualizes how specific skin phenotypes inform early differential diagnosis remains lacking. In particular, existing literature does not systematically address whether distinct or atypical cutaneous patterns—such as annular, vesiculobullous, psoriasiform, erythema nodosum–like lesions, or *Bacillus Calmette–Guérin* (BCG) scar reactivation—can function as diagnostic modifiers in incomplete or overlapping presentations of KD and MIS-C. Accordingly, the primary aim of this narrative review is to address this gap by critically synthesizing available evidence to answer the following research question: can the morphology, distribution, and temporal evolution of cutaneous manifestations meaningfully contribute to the early differentiation between KD and MIS-C in pediatric patients with persistent fever and systemic inflammation? By organizing heterogeneous dermatologic presentations into a comparative, clinically oriented framework, this review seeks to move beyond a purely descriptive approach and to provide clinicians with pragmatic interpretative cues that may support earlier recognition, diagnostic stratification, and timely management of these high-risk inflammatory syndromes.

## 2. Methods

A comprehensive literature search was conducted using the PubMed database to identify published studies describing cutaneous manifestations associated with Kawasaki disease (KD) and multisystem inflammatory syndrome in children (MIS-C). The search strategy combined Medical Subject Headings (MeSH) terms and free-text keywords, including “Kawasaki disease,” “multisystem inflammatory syndrome in children,” “MIS-C,” “cutaneous,” “skin,” “rash,” and “mucocutaneous”, using Boolean operators as appropriate.

The search encompassed articles published between January 2006 and December 2025. This time frame was selected to ensure greater methodological and clinical consistency across included studies, as publications from this period more commonly apply contemporary KD diagnostic criteria, standardized echocardiographic assessment of coronary involvement, and current immunomodulatory treatment approaches. In addition, restricting the search to this interval allowed meaningful comparison with MIS-C, which emerged only after 2020, while still capturing modern KD literature with detailed dermatologic descriptions.

To ensure relevance to the pediatric population, eligibility was restricted to studies involving individuals aged 0–18 years. Only articles published in English were considered. All retrieved records were initially screened based on title and abstract to assess relevance, followed by full-text review when abstracts suggested potential eligibility.

Studies were included if they reported clinical, epidemiological, or dermatological features of KD or MIS-C with sufficiently detailed descriptions of cutaneous or mucocutaneous manifestations to allow clinical interpretation. Eligible study designs included case reports, case series, and observational studies, reflecting the rarity, heterogeneity, and often atypical nature of dermatologic presentations in both conditions. Editorials, commentaries, narrative reviews, conference abstracts, and studies in which skin findings were only marginally mentioned or insufficiently characterized were excluded.

The study selection process was supported by the Rayyan web-based systematic review platform, which facilitated duplicate removal and independent, blinded screening of records by multiple reviewers. Discrepancies were resolved through discussion and consensus. Although the inclusion criteria were intentionally broad, many screened articles were excluded because they lacked detailed dermatologic characterization, used non-specific terminology, or did not allow differentiation between KD- and MIS-C-related skin findings. Consequently, the final selection comprised a limited number of studies that were directly informative for the study aim.

Given the heterogeneity of study designs, descriptive reporting of outcomes, and absence of standardized definitions or quantitative measures for cutaneous manifestations, a quantitative synthesis or meta-analysis was not feasible. Therefore, findings were synthesized narratively, with cutaneous manifestations categorized according to morphology, distribution, temporal evolution, and clinical context to enable a structured and clinically oriented comparison between KD and MIS-C.

## 3. Pictorial Description of Skin Manifestations in Kawasaki Disease and Multisystem Inflammatory Syndrome in Children

Several reports have highlighted the broad and often heterogeneous spectrum of cutaneous manifestations associated with KD [12,13,14,15]. Ming et al. described three pediatric patients presenting with annular lesions, some exhibiting a targetoid appearance that initially involved the hands and feet and later extended to the trunk [16]. These lesions were frequently accompanied by classical features of KD, including edema of the extremities and fissured lips. In one 5-year-old patient, widespread annular lesions extended from the lower limbs to the perineal region and were associated with bilateral scrotal erythema, conjunctivitis, and myocardial involvement. The patient required treatment with intravenous immunoglobulin (IVIG), inotropic support, and subsequent cardiac therapy [16]. Another case involved a 3-year-old boy with annular lesions initially confined to the thighs and later spreading to the hands, feet, and perineum, associated with nailbed erythema and conjunctivitis; he responded favorably to IVIG and aspirin. In a third patient, a 2-year-old girl, annular and scaly eruptions with pustular elements affected the trunk and limbs, accompanied by edema of the hands and feet and splinter hemorrhages at the nail beds. Following failure of antibiotic therapy, IVIG led to clinical resolution, although streptococcal coinfection was later confirmed serologically [16].

Additional distinctive presentations included inflammatory reactions at BCG vaccination sites, a phenomenon described in countries where neonatal BCG vaccination is routine. In one case, a 14-month-old boy developed erythema, swelling, and ulceration at the vaccination site, initially misdiagnosed as cellulitis or thrombophlebitis. Imaging excluded deep tissue infection, and clinical improvement occurred only after IVIG administration, alongside the later development of coronary artery dilation, confirming KD [17]. A second infant exhibited generalized erythematous rash, perineal involvement, conjunctival injection, and marked inflammation at the BCG inoculation site, with resolution following immunoglobulin and corticosteroid therapy [18].

Several reports describe patients presenting with more typical maculopapular eruptions. In one case, a 17-month-old boy developed diffuse pruritic and painful eruptions involving the scalp, trunk, and extremities, later complicated by vesicular lesions and coronary artery dilation, necessitating repeated IVIG infusions and corticosteroid therapy [19]. Another case involved a 2-year-old boy with a papular eruption initially confined to the trunk and later extending to the diaper area, accompanied by genital edema; both responded to IVIG and aspirin [20].

Erythema nodosum-like lesions have also been described as atypical manifestations of KD. A 3-year-old girl presented with fever, painful nodules on the lower extremities, and cervical lymphadenopathy. Initial misdiagnosis as erythema nodosum delayed recognition of KD until coronary artery aneurysms were identified, underscoring the diagnostic challenge posed by overlapping dermatologic phenotypes [21]. Similarly, jaundice-associated KD has been reported in several cases, where hepatic involvement and hyperbilirubinemia initially suggested infectious hepatitis or sepsis, delaying appropriate diagnosis and treatment [22,23,24,25,26,27,28].

Age-related variability further complicates diagnosis. Infants younger than 6 months and adolescents often present with incomplete or atypical features, increasing the risk of delayed diagnosis and coronary complications [29,30,31,32,33,34,35,36,37]. For example, a 3-month-old infant with minimal initial symptoms developed coronary aneurysms within days despite early intervention, highlighting the aggressive nature of KD in very young children [38]. Conversely, adolescents may present with prolonged fever, mucocutaneous findings, and systemic symptoms that overlap with infectious or autoimmune diseases, often delaying diagnosis.

Several reports also describe psoriasiform and vesiculobullous eruptions associated with KD. A 12-month-old child developed diffuse erythematous plaques following acute KD, later confirmed as psoriasis by histopathology [39]. Another infant developed severe vesiculobullous lesions with subsequent arthritis and coronary involvement, eventually responding to infliximab after failure of standard therapies [40]. These cases underscore the potential for KD to trigger immune-mediated dermatologic conditions, possibly through immune dysregulation mechanisms.

Finally, overlapping features between KD and MIS-C have been increasingly recognized at the onset of the description of MIS-C. Reports describe patients with fever, mucocutaneous inflammation, conjunctivitis, rash, and systemic involvement mimicking KD but occurring in the context of prior SARS-CoV-2 exposure. In several cases, extensive rashes—ranging from morbilliform to urticarial and vasculitic—were prominent, and laboratory findings revealed marked inflammation, lymphopenia, and cardiac involvement. These observations reinforce the concept that MIS-C and KD share overlapping immunopathogenic pathways, with cutaneous manifestations representing a key diagnostic clue [41,42].

Table 1 shows the comparison of clinical and epidemiological features of KD and MIS-C, whereas Table 2 compares cutaneous manifestations in KD and MIS-C.

Collectively, cutaneous manifestations associated with KD and MIS-C encompass a wide and well-described spectrum of morphologies that can be broadly categorized according to clinical pattern rather than individual case presentations. In KD, reported skin findings include polymorphous exanthema, acral erythema and edema, periungual desquamation, perineal erythema, and less typical patterns such as annular or targetoid lesions, vesiculobullous eruptions, psoriasiform plaques, erythema nodosum–like nodules, and inflammatory reactivation at BCG vaccination sites. MIS-C displays similarly heterogeneous cutaneous involvement, most commonly presenting with morbilliform, scarlatiniform, urticarial, reticulated, or vasculitic-appearing rashes, frequently accompanied by conjunctival injection and mucosal inflammation. Importantly, the substantial overlap between KD- and MIS-C–associated skin phenotypes was recognized from the earliest descriptions of MIS-C and remains a major diagnostic challenge. For this reason, rash morphology alone is insufficient for differentiation and must be interpreted within the broader clinical context, including patient age, systemic involvement, inflammatory markers, and evidence of recent or prior SARS-CoV-2 exposure.

## 4. Cutaneous Heterogeneity in Kawasaki Disease and MIS-C: Diagnostic Challenges and Clinical Implications

The cases analyzed illustrate how skin findings—often among the earliest and most visible manifestations—can range from classic to highly atypical, potentially delaying recognition and treatment. Several reports described patients presenting with annular or targetoid lesions, frequently beginning on acral sites and later spreading to the trunk or perineum. These patterns, often accompanied by acral edema, fissured lips, and bilateral non-exudative conjunctivitis, reflect well-recognized features of KD but may initially be mistaken for other dermatologic entities. In some cases, annular or pustular lesions and splinter hemorrhages led to initial misdiagnoses such as Stevens–Johnson syndrome or infectious dermatoses, emphasizing the protean nature of KD-related skin involvement. The occurrence of scrotal erythema, nail-bed changes, and perineal involvement further underscores the need for heightened clinical suspicion, particularly when systemic inflammation or cardiac involvement is present.

A particularly notable feature described in several cases was inflammation at the BCG inoculation site, ranging from erythema and induration to ulceration. Although not pathognomonic, BCG site reactivation is increasingly recognized as a valuable diagnostic clue in infants with KD, especially in countries where universal BCG vaccination is practiced. When present alongside fever and mucocutaneous findings, this sign should prompt immediate evaluation for KD.

The reviewed literature also highlights cases in which KD manifested with predominantly maculopapular or vesiculobullous eruptions, sometimes progressing to psoriasiform or pustular patterns. These atypical presentations often led to delayed diagnosis, as they mimicked infectious exanthems, drug reactions, or inflammatory dermatoses. Notably, several reports described the evolution of psoriasis-like eruptions following acute KD, suggesting that immune dysregulation during the disease course may trigger or unmask chronic inflammatory skin conditions in genetically predisposed individuals.

Erythema nodosum–like lesions represent another diagnostic pitfall. In several cases, patients initially diagnosed with erythema nodosum were later found to have KD after the development of coronary artery abnormalities. These observations highlight the importance of considering KD in the differential diagnosis of children presenting with panniculitis, particularly when systemic inflammation persists despite appropriate antimicrobial therapy.

Age-related variability further complicates recognition. Infants younger than six months and older children or adolescents often present with incomplete or atypical forms of KD, lacking the full constellation of diagnostic criteria. In these age groups, cutaneous findings may be subtle, atypical, or transient, yet the risk of coronary involvement remains substantial. Consequently, clinicians must maintain a high index of suspicion even in the absence of classic features.

The overlap between KD and MIS-C adds another layer of diagnostic complexity. Increasing evidence indicates that MIS-C can mimic KD both clinically and dermatologically, with shared features such as polymorphic rashes, conjunctivitis, mucosal changes, and acral edema. However, MIS-C often presents with more prominent systemic inflammation, gastrointestinal involvement, and myocardial dysfunction. Reports of MIS-C cases with urticarial, vasculitic, or polymorphic eruptions highlight the importance of recognizing these patterns as part of a post-infectious hyperinflammatory syndrome rather than isolated dermatologic disease.

Collectively, these observations underscore that cutaneous manifestations in KD and MIS-C are highly variable and may evolve rapidly over time. Recognizing atypical presentations—such as annular or psoriasiform lesions, erythema nodosum–like nodules, BCG site reactivation, or vesiculobullous eruptions—is essential for early diagnosis and timely intervention. Given the potential for severe cardiovascular complications, clinicians should maintain a high index of suspicion for KD and MIS-C in any child presenting with persistent fever and unexplained skin findings, even when classical diagnostic criteria are not fully met.

## 5. Interpreting Cutaneous Patterns Within Kawasaki Disease and MIS-C: Diagnostic Algorithms

Cutaneous manifestations play a different diagnostic role within the established frameworks for KD and MIS-C, acting as core criteria, supportive elements, or context-dependent modifiers depending on their morphology and clinical setting. In classic KD, a polymorphous rash represents one of the five principal diagnostic criteria and may include maculopapular, morbilliform, urticarial, or scarlatiniform patterns. When these typical eruptions are accompanied by acral erythema or edema, bilateral non-exudative conjunctivitis, and mucosal changes, they contribute directly to fulfilling the criteria for complete KD. In contrast, atypical morphologies—such as annular, targetoid, vesiculobullous, psoriasiform, or erythema nodosum–like lesions—still satisfy the rash criterion but frequently divert patients toward an incomplete KD diagnostic pathway, particularly when other classical signs are absent or delayed.

In incomplete KD, cutaneous findings acquire increased diagnostic relevance when integrated with supportive laboratory markers of systemic inflammation and echocardiographic abnormalities. Certain dermatologic features, although not included in formal criteria, may act as strong disease indicators in this context. Notably, inflammation or reactivation at the BCG vaccination site, periungual desquamation during the subacute phase, and perineal or genital involvement are highly suggestive of KD and may prompt early diagnostic confirmation despite incomplete clinical presentation. Conversely, erythema nodosum–like lesions or vesiculobullous eruptions may initially obscure the diagnosis, leading to misclassification as infectious or immune-mediated dermatoses and delaying recognition of KD.

Within the case definitions proposed by the Centers for Disease Control and Prevention, the World Health Organization, and the European Centre for Disease Prevention and Control, cutaneous involvement is framed more broadly as part of mucocutaneous inflammation and does not require a specific morphology. Morbilliform, urticarial, vasculitic, reticulated, or polymorphic rashes all contribute to fulfilling MIS-C criteria when associated with persistent fever, laboratory evidence of hyperinflammation, multisystem involvement, and documented or probable exposure to SARS-CoV-2. In this setting, urticarial or vasculitic-appearing eruptions, diffuse mucositis, and conjunctival injection are frequently reported and may carry greater diagnostic weight when accompanied by gastrointestinal symptoms, myocardial dysfunction, or shock.

Importantly, overlapping cutaneous phenotypes between KD and MIS-C highlight the need for contextual interpretation rather than reliance on rash morphology alone. While periungual desquamation and BCG scar reactivation strongly favor KD, extensive urticarial or vasculitic rashes in older children with marked systemic inflammation are more consistent with MIS-C definitions. Thus, cutaneous patterns serve as diagnostic anchors in classic KD, as amplifiers in incomplete KD, and as supportive but non-specific features in MIS-C, reinforcing the importance of integrating dermatologic findings into a broader clinical, laboratory, and epidemiological framework.

Previous publications have described the overlap between KD and MIS-C with respect to mucocutaneous involvement, often focusing on selected clinical signs or illustrative examples [12,13,14,15]. In contrast, the present review provides a broader and more structured synthesis of the full spectrum of cutaneous manifestations reported in both conditions, with particular emphasis on atypical and diagnostically challenging presentations. Rather than reiterating individual findings, this work integrates skin morphology, distribution, temporal evolution, and clinical context, and explicitly maps these features onto current diagnostic frameworks for classic and incomplete Kawasaki disease and contemporary definitions of multisystem inflammatory syndrome in children. By doing so, the review clarifies how dermatologic findings may function as diagnostic modifiers that influence clinical reasoning, risk stratification, and therapeutic decision-making, thereby addressing a gap not systematically explored in earlier descriptive or pictorial reports.

## 6. Conclusions

Beyond summarizing existing reports, this review contributes to the literature by providing a structured, clinically oriented synthesis of cutaneous manifestations in KD and MIS-C, with specific emphasis on atypical and underrecognized presentations. By mapping heterogeneous skin phenotypes onto established diagnostic frameworks for classic and incomplete KD and current MIS-C definitions, this work highlights how dermatologic findings can function as diagnostic modifiers rather than isolated signs. The review further underscores the clinical relevance of certain patterns—such as annular, vesiculobullous, psoriasiform, erythema nodosum-like lesions, and BCG scar reactivation—that are frequently reported but inconsistently interpreted in prior literature. In doing so, it offers a pragmatic interpretative framework to support earlier recognition, risk stratification, and timely escalation of immunomodulatory therapy in children presenting with persistent fever and atypical cutaneous findings.

Diagnosis of KD is based on a constellation of clinical criteria, and patients who do not fulfill the complete diagnostic framework may still develop incomplete KD, with a delay in diagnosis and a high risk of coronary artery involvement [25,43]. This risk is particularly relevant in infants younger than six months, who often present with prolonged unexplained fever and few or absent classical signs, making early diagnosis especially challenging [3]. MIS-C represents another serious inflammatory condition, emerging as a post–SARS-CoV-2 complication with an incidence of 2 per 100,000 SARS-CoV-2 persons younger than 21 years previously infected with SARS-CoV-2 [44]. Cutaneous manifestations are among the most frequent clinical features of MIS-C, occurring in nearly three-quarters of cases, and often represent one of the earliest signs of disease onset [42].

Atypical cutaneous presentations may substantially influence both the timing and intensity of therapeutic interventions in KD and MIS-C. When skin manifestations deviate from the classic polymorphous exanthema—such as annular, vesiculobullous, psoriasiform, or erythema nodosum–like lesions—diagnostic uncertainty is common, often leading to initial misclassification as infectious, allergic, or drug-related dermatoses. This diagnostic delay can postpone first-line immunomodulatory treatment, particularly IVIG, which is most effective when administered early in the disease course to reduce the risk of coronary artery involvement. Moreover, atypical dermatologic phenotypes may reflect heightened or dysregulated immune activation and have been associated with IVIG resistance, thereby necessitating earlier consideration of second-line therapies such as systemic corticosteroids or biologic agents, including tumor necrosis factor or interleukin-1 inhibitors. In the context of MIS-C, extensive or vasculitic-appearing rashes may prompt more rapid escalation to combination therapy with IVIG and corticosteroids and closer cardiovascular monitoring. Consequently, heightened awareness of atypical cutaneous patterns is essential, as their recognition may not only accelerate diagnostic confirmation but also inform risk stratification and timely escalation of immunomodulatory treatment, ultimately improving clinical outcomes.

Recognition of skin manifestations suggestive of KD or MIS-C is therefore crucial, as these findings frequently precede or accompany systemic involvement. Early identification enables timely initiation of immunomodulatory therapy, which is essential for preventing severe complications—particularly cardiovascular sequelae. As highlighted throughout this review, the spectrum of cutaneous involvement is remarkably broad, ranging from classic erythematous and polymorphous rashes to psoriasiform, vesicular, urticarial, and erythema nodosum–like lesions. In some cases, jaundice-like skin discoloration or other atypical manifestations may further complicate the clinical picture.

The marked heterogeneity of these dermatologic findings underscores the diagnostic challenge posed by both KD and MIS-C. Because such manifestations may overlap with infectious, allergic, or autoimmune conditions, they are often insufficient on their own to distinguish between the two syndromes. Consequently, a high index of suspicion is required, particularly when persistent fever coexists with mucocutaneous involvement. In this context, evidence of cardiac involvement and documentation of recent or prior SARS-CoV-2 infection can provide critical diagnostic clues. Ultimately, heightened awareness of the diverse cutaneous presentations associated with KD and MIS-C is essential to facilitate early diagnosis, guide appropriate management, and reduce the risk of potentially life-threatening complications.

## Figures and Tables

**Table 1:** Comparison of Clinical and Epidemiological Features of Kawasaki Disease and MIS-C.

Feature	Kawasaki Disease (KD)	MIS-C
Typical age	<5 years	School-aged children and adolescents
Trigger	Unknown; likely infectious	Post–SARS-CoV-2 infection
Onset	Acute	Delayed (2–6 weeks post-infection)
Fever	Persistent ≥ 5 days	Persistent, often high-grade
Cardiac involvement	Coronary artery aneurysms	Myocarditis, shock
Gastrointestinal symptoms	Occasional	Common
Laboratory findings	Elevated ESR/CRP, thrombocytosis	High CRP, ferritin, D-dimer
Response to IVIG	Usually good	Often requires steroids/biologics

CRP, C reactive protein; ESR, erythrocyte sedimentation rate; IVIG, intravenous immunoglobulin.

**Table 2:** Cutaneous Manifestations in KD and MIS-C.

Cutaneous Feature	KD	MIS-C
Polymorphous rash	Common	Common
Annular/targetoid lesions	Rare	Rare
Periungual desquamation	Common	Rare
Mucosal involvement	Common	Common
BCG scar reactivation	Common	Rare
Psoriasiform eruptions	Rare	Rare
Urticarial/vasculitic lesions	Rare	Common

MIS-C, multisystem inflammatory syndrome in children; KD, Kawasaki disease.

## Data Availability

All the available data are included in the manuscript.
